# CAMF-DTI: Enhancing Drug–Target Interaction Prediction via Coordinate Attention and Multi-Scale Feature Fusion

**DOI:** 10.3390/cimb47110964

**Published:** 2025-11-20

**Authors:** Jia Mi, Chang Li, Daguang Jiang, Jing Wan

**Affiliations:** 1 College of Information Science and Technology, Beijing University of Chemical Technology, Beijing 100029, China; 2023400222@buct.edu.cn (J.M.); 1994500057@buct.edu.cn (D.J.); 2 School of Computer Science and Technology, Harbin Institute of Technology, Harbin 150001, China; cli@ir.hit.edu.cn

**Keywords:** drug–target interaction, coordinate attention, multi-scale feature fusion, cross-attention

## Abstract

The accurate prediction of drug–target interactions is essential for drug discovery and development. However, current models often struggle with two challenges. First, they fail to model the directional flow and positional sensitivity of protein sequences, which are critical for identifying functional interaction regions. Second, they lack mechanisms to integrate multi-scale information from both local binding sites and broader structural context. To overcome these limitations, we propose CAMF-DTI, a novel framework that incorporates coordinate attention, multi-scale feature fusion, and cross-attention to enhance both the representation and interaction learning of drug and protein features. Drug molecules are represented as molecular graphs and encoded using graph convolutional networks, while protein sequences are processed with coordinate attention to preserve directional and spatial information. Multi-scale fusion modules are applied to both encoders to capture local and global features, and a cross-attention module integrates the representations to enable dynamic drug–target interaction modeling. We evaluate CAMF-DTI on four benchmark datasets: BindingDB, BioSNAP, C.elegans, and Human. Experimental results show that CAMF-DTI consistently outperforms seven state-of-the-art baselines in terms of AUROC, AUPRC, Accuracy, F1-score, and MCC. Ablation studies further confirm the effectiveness of each module, and visualization results demonstrate the model’s potential interpretability.

## 1. Introduction

Accurately identifying potential drug–target interactions (DTIs) is a central step in the early stages of drug development, enabling efficient drug discovery, repurposing, and safety evaluation [1,2,3]. However, traditional experimental methods such as high-throughput screening and affinity assays are often time-consuming, expensive, and limited in scale. In this context, computational DTI prediction has become not only a promising complement to experimental methods but an urgent necessity [4,5].

Deep learning-based DTI prediction models have advanced significantly in recent years, offering scalable, end-to-end solutions for modeling complex drug–target relationships. Broadly, these models can be categorized into two main classes: sequence-based and structure-based approaches [6]. Sequence-based methods focus on one-dimensional molecular representations, such as SMILES strings for drugs and amino acid sequences [7] for proteins. Convolutional neural networks, as used in DeepDTA [8] and WideDTA [9], are employed to extract local patterns from these sequences. Transformer-based architectures, including Trans formerCPI [10] and MolTrans [11], further enhance sequence modeling by capturing long-range dependencies within molecular sequences. Structure-based methods incorporate topological and spatial features of molecular structures to improve representation learning. Graph-based approaches, such as GraphDTA [12], MGraphDTA [13], and NeoDTI [14], treat drug compounds as molecular graphs and apply graph neural networks to capture local connectivity and substructural patterns. Other models, such as GraphDTA [12], Graph-DTI [15], and EquiBind [16], go beyond 2D topology by integrating residue-level 3D coordinates or pairwise distance matrices to represent protein spatial conformation [17]. These structure-aware designs enable the modeling of geometric compatibility and physicochemical interactions at binding interfaces. These models have achieved competitive results on benchmark datasets such as BindingDB [18], Davis [19], and KIBA [20], highlighting the important roles of sequence-derived and structure-enriched representations in DTI prediction.

Despite recent progress, existing DTI models still face core limitations in both sequence and structure modeling. In sequence-based models, a major problem is the lack of interactive representation learning. Drugs and proteins are often encoded independently and fused only at the final stage, preventing the model from capturing dynamic dependencies between them. In structure-based models, two limitations are common. (1) Most methods ignore the directional nature [21] of protein structures, such as the N-to-C terminal flow, making it hard to distinguish regions with similar geometry but different biological roles. (2) Many models use single-scale aggregation strategies, which limit their ability to capture both local binding sites and global conformational features [22,23].

To address the above issues, this study proposes a DTI prediction model named CAMF DTI, which integrates coordinate attention, multi-scale feature fusion, and cross-attention mechanisms. This architecture enhances the modeling of complex structural and interaction patterns between drugs and proteins. The main contributions are as follows:

- To tackle the lack of directional information in spatial features, coordinate attention is introduced to jointly encode spatial position and sequence directionality, improving the localization of key interaction regions.
- To address the limitation of single-scale receptive fields, a multi-scale fusion module is designed to extract structural features at different scales via parallel branches, enabling unified representation of local and global information.
- To overcome insufficient modeling of drug–target interactions, a cross-attention mechanism is employed to capture dynamic dependencies between drugs and targets during representation learning.
- Experimental results on multiple benchmark datasets demonstrate that CAMF-DTI outperforms existing state-of-the-art methods across AUROC, AUPRC, Accuracy, F1 score, and MCC metrics, indicating superior predictive performance and strong generalization ability.

## 2. Materials and Methods

### 2.1. The Architecture of CAMF-DTI

**Figure 1:**
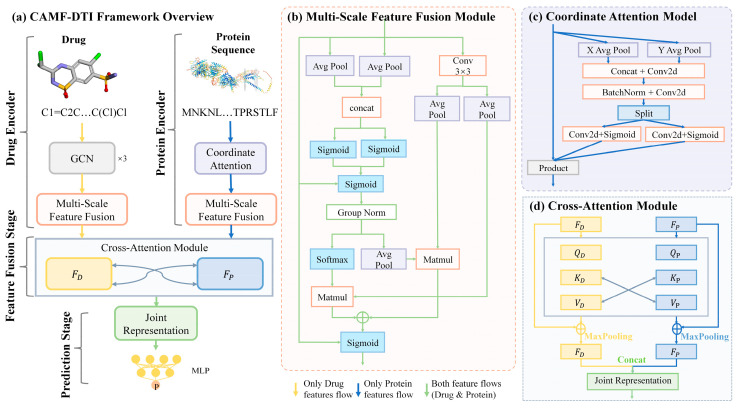
CAMF-DTI framework diagram. (**a**) CAMF-DTI Framework Overview; (**b**) Multi-Feature Fusion Module; (**c**) Coordinate Attention Model; (**d**) Cross-Attention Module.

The overall architecture of the proposed model CAMF-DTI is illustrated in Figure 1. The framework consists of several components, including a drug encoder, a protein encoder, a feature fusion stage, and a prediction stage. Among them, three key modules are specifically designed to address the core limitations of existing methods. Specifically, drug molecules represented by SMILES [7] strings are first processed by a three-layer graph convolutional network (GCN) [24] to extract structural and substructural features. Protein sequences are then passed through the Coordinate Attention Module, which encodes spatial information while incorporating sequence directionality, thereby highlighting functionally relevant regions. Subsequently, both drug and protein features are refined using the Multi-Scale Feature Fusion Module, which captures local binding patterns and global conformational information at multiple receptive fields to achieve multi-level feature representation. Finally, the Cross-Attention Module models dynamic interactions between drugs and proteins, generating a joint representation that is passed to multilayer perceptrons (MLPs) for the final DTI prediction.

### 2.2. Drug Encoder

#### 2.2.1. Drug Feature Construction

In drug feature extraction, the SMILES sequence is converted into a molecular graph *G* = (*V*, *E*), where *V* denotes atom nodes and *E* denotes chemical bonds. Using the DGL-LifeSci toolkit (version 1.0; AWS, USA), each atom is encoded as a 74-dimensional feature vector (atom type, degree, hydrogen count, charge, hybridization, aromaticity, etc.). The detailed atom-level features used in the molecular graph construction are summarized in Table S1 (Supplementary Materials). The node feature matrix of each molecule is denoted as $M_{d}\in {\mathbb{R}}^{N_{d}\times 74}$, where *N_d_* is the maximum number of atoms, and zero-padding is applied if necessary.

A linear transformation is applied to obtain dense input features:

(1) $$X_{d}=M_{d}W_{d}^{T}$$

#### 2.2.2. GCN

To learn molecular representations from the drug graph, we employ a three-layer Graph Convolutional Network (GCN). At layer *l*, the node feature update is defined as:

(2) $$H_{d}^{\left(l+1\right)}=\sigma \left(\tilde{A}H_{d}^{\left(l\right)}W_{g}^{\left(l\right)}+b_{g}^{\left(l\right)}\right)$$

where $H_{d}^{\left(l\right)}$ is the node feature matrix, $\tilde{A}$ is the adjacency matrix (with self-connections), $W_{g}^{\left(l\right)}$ and $b_{g}^{\left(l\right)}$ denote the weight and bias of the l-th GCN layer, and σ represents the activation function. 

This process aggregates local substructures and captures global chemical information, producing high-level molecular graph representations for downstream fusion.

#### 2.2.3. Multi-Scale Feature Fusion

To further enhance the structural representation learned from the GCN-based drug encoder, we introduce a multi-scale feature fusion module inspired by the model proposed by Ouyang [25] (as illustrated in Figure 1b). This module is designed to capture and integrate local and global features across different receptive fields, thereby improving the expressiveness and robustness of learned representations. Notably, the same module is also incorporated in the protein encoder, allowing both drug and protein features to be refined with rich multi-scale spatial and semantic information.

Let the input be denoted as:

(3) $$X=[X_{0},\;X_{1},\ldots ,X_{G-1}]$$

where each group $X_{i}\in {\mathbb{R}}^{\frac{C}{G}\times H\times W}$ is processed through three parallel branches: two with 1 × 1 convolutions to model local channel-wise dependencies, and one with a 3 × 3 convolution to capture spatial context.

To encode global-level semantics, two average pooling operations are applied across orthogonal directions. Their outputs are concatenated and passed through multiple Sigmoid and GroupNorm layers, enhancing spatial alignment. These aggregated signals are then normalized and passed through a Softmax operation to generate scale-wise attention weights:

(4) $$Z_{c}=\frac{1}{HW}\sum_{i=1}^{H}\sum_{j=1}^{W}X_{c}(i,\;j)$$

where *Z*_C_ is the pooled descriptor for channel *c*. These descriptors are normalized and passed through a softmax function to compute attention weights across scales. Weighted aggregation is then performed, followed by sigmoid activation to obtain the final fused representation.

By combining local and global features across multiple receptive fields, the multi-scale feature fusion module provides a more expressive and robust feature representation for downstream interaction modeling. Notably, this module is also employed in the protein encoder with the same architecture and processing pipeline, where it is applied after the coordinate attention mechanism to ensure consistent enhancement of hierarchical semantic representations from both modalities. This parallel application maintains architectural coherence between the two encoders while allowing flexible adaptation to their distinct data characteristics.

Although both the drug and protein encoders adopt the same multi-scale feature fusion architecture for consistency, these modules are jointly trained within the end-to-end optimization process of CAMF-DTI. This joint training enables the model to learn modality-specific patterns while promoting coordinated feature evolution between the two encoders. The gradients from the interaction and prediction stages are backpropagated through both encoders simultaneously, ensuring that the multi-scale fusion modules are optimized collaboratively to enhance the complementarity of drug and protein representations.

### 2.3. Protein Encoder

#### 2.3.1. Protein Feature Construction

To enhance the protein representation and capture long-range dependencies within amino acid sequences, we first initialize each protein sequence *P* by looking up a learnable embedding matrix $E_{p}\in {\mathbb{R}}^{22\times D_{p}}$, where 22 is the number of standard amino acid types and *D_p_* is the embedding dimension. Here, the term “long-range dependency” refers to intra-sequence contextual relationships among distant amino acid residues rather than intermolecular interactions. Modeling such dependencies enables the network to capture non-local structural and functional correlations within the protein chain.

The resulting sequential feature matrix $X_{p}\in {\mathbb{R}}^{\theta_{p}\times D_{p}}$ (with $\theta_{p}$ being the maximum sequence length) is then passed through a multi-order gated convolution encoder, which recursively captures hierarchical and multi-scale contextual information across the sequence.

#### 2.3.2. Coordinate Attention Mechanism

To effectively capture the long-range dependencies and spatially significant positions within protein sequences, we incorporate a coordinate attention mechanism inspired by the model proposed by Hou [26] (as illustrated in Figure 1c). This design addresses the limitations of traditional convolutional architectures, which often fail to model global context and positional sensitivity, especially for long amino acid chains. The coordinate attention mechanism enhances the model’s ability to locate and emphasize informative positions by embedding directional and positional information into the feature maps.

To enable the application of the coordinate attention module, the output of the encoder, originally in the form of a sequential matrix $X_{p}\in {\mathbb{R}}^{\theta_{p}\times D_{p}}$, is reshaped into a 3D tensor $x\in {\mathbb{R}}^{C\times H\times W}$. Here, *C* denotes the number of feature channels, with *D_p_* equal to *C*, and *H* and *W* are spatial dimensions such that $H\times W=\theta_{p}$.

Specifically, for a given protein input $x\in {\mathbb{R}}^{C\times H\times W}$, two pooling operations are applied along the horizontal and vertical directions to encode direction-aware contextual information. The aggregated features are computed as:

(5) $$z_{c}^{h}(h)=\frac{1}{W}\sum_{0\leq i<W}x_{c}(h,\;i)$$

(6) $$z_{c}^{\omega }(\omega )=\frac{1}{H}\sum_{0\leq j<H}x_{c}(j,\;\omega )$$

These features are concatenated and passed through a shared 1 × 1 convolution to form an intermediate representation. This is then split and transformed by two separate 1 × 1 convolutions with learnable parameters $F_{h}$ and $F_{\omega }$, yielding the attention weights.

(7) $$g^{i}=\sigma \left(F_{i}(f^{i})\right),\;i=h,\omega$$

where σ denotes the sigmoid activation function. Finally, the output of the coordinate attention block is given by:

(8) $$y_{c}(i,\;j)=x_{c}(i,\;j)\times g_{c}^{h}(i)\times g_{c}^{\omega }(j)$$

Notably, the output of this module is subsequently passed into the Multi-Scale Feature Fusion Module to further enrich the representation by integrating features from multiple receptive fields.

In contrast to conventional attention mechanisms that focus solely on channel-wise dependencies, the coordinate attention mechanism decomposes channel attention into two direction-aware branches, enabling the model to encode both spatial position and sequence directionality. For protein sequences, the N-to-C terminal order often implies structural and functional asymmetry. By aggregating contextual information along horizontal and vertical directions, this mechanism guides the network to emphasize biologically meaningful regions, such as binding pockets or active sites, thereby improving the localization and interpretability of protein representations.

### 2.4. Feature Fusion Stage

To fuse the hierarchical features of drugs and proteins and accurately capture their interaction patterns, we introduce a cross-attention mechanism. This module adaptively highlights key regions of both representations by treating one modality as query and the other as key-value pairs, enhancing the discriminability of interaction features. The structure is illustrated in Figure 1d.

#### 2.4.1. Interaction Feature Extraction Module

Let the output features from the drug and protein encoders be denoted as *F_D_* and *F_P_*, respectively. The query and key representations are computed as follows:

(9) $$Q_{D}=F_{D}W_{D}^{q},\;K_{p}=F_{p}W_{p}^{k}$$

(10) $$Q_{p}=F_{p}W_{p}^{q},\;K_{D}=F_{D}W_{D}^{k}$$

where $W^{q}$ and $W^{k}$ are modality-specific projection matrices.

Cross-attention is then applied to generate interaction-aware representations via scaled dot-product attention:

(11) $$Z_{D}=\mathrm{Softmax}\left(\frac{Q_{D}K_{p}^{T}}{\sqrt{d_{k}}}\right)$$

(12) $$Z_{p}=\mathrm{Softmax}\left(\frac{Q_{p}K_{D}^{T}}{\sqrt{d_{k}}}\right)$$

$Z_{D}$ and $Z_{P}$ are the attention weights obtained by applying the Softmax function to the scaled dot-products between the queries and keys.

The fused interaction-aware representations are computed by integrating attention weights.

(13) $$D_{f}=0.5\cdot Z_{D}+0.5\cdot F_{D}$$

(14) $$P_{f}=0.5\cdot Z_{p}+0.5\cdot F_{p}$$

$D_{f}$ and $P_{f}$ are the interaction-aware fused representations of drugs and proteins, respectively, computed as the weighted average of attention-aware features and the original features.

To obtain fixed-length representations, we apply global max pooling:

(15) $$D_{f}^{r}=\mathrm{MaxPooling}(D_{f})$$

(16) $$P_{f}^{r}=\mathrm{MaxPooling}(P_{f})$$

Finally, we concatenate the pooled features to form the unified interaction representation:

(17) $$f=\mathrm{concat}(D_{f}^{r},\;P_{f}^{r})$$

#### 2.4.2. Prediction Module

To predict the probability of DTI, the fused feature representation is fed into a decoder composed of fully connected layers. The predicted DTI probability *p* is computed as:

(18) p=σ(Wf+b)

where *W* and *b* are learnable weight matrix and bias vector, respectively.

During model training, the parameters are optimized using backpropagation. The loss function employed is the cross-entropy loss function, defined as:

(19) $$\mathrm{loss}=-\sum_{i}\left(y_{i}\log(p_{i})+(1-y_{i})\log(1-p_{i})\right)$$

where $y_{i}$ denotes the ground-truth label for the *i*-th drug–target pair, and *p*_*i*_ represents the predicted DTI probability.

## 3. Results and Discussion

### 3.1. Evaluation Metrics

To better evaluate the performance of the model, we adopt AUROC (Area Under the Receiver Operating Characteristic Curve) [27], AUPRC (Area Under the Precision-Recall Curve) [27], F1-Score [28], Accuracy [29], and MCC (Matthews Correlation Coefficient) as the evaluation metrics across the four benchmark datasets. Among them, MCC is a metric specifically designed for evaluating binary classification performance. It considers all elements of the confusion matrix, including true positives, true negatives, false positives, and false negatives, and is regarded as a relatively balanced measure.

### 3.2. Datasets

We evaluate our model on four public datasets: BindingDB, BioSNAP, Human, and C.elegans. BindingDB is a large-scale dataset of experimentally verified drug–target interactions, containing 14,643 drugs, 2623 proteins, and 49,199 interactions. BioSNAP is a balanced dataset derived from DrugBank, with 4510 drugs, 2181 proteins, and 27,464 interactions. Human includes 2726 drugs and 2001 proteins, covering 6728 balanced interaction pairs. C.elegans shares the same scale as the Human dataset but focuses on interactions specific to the C.elegans species. The detailed statistics are summarized in Table 1.

### 3.3. Baseline Methods

To validate the effectiveness of CAMF-DTI, we conducted experiments on four datasets: BindingDB, BioSNAP, C.elegans, and Human. Seven representative baseline methods were selected for comparison:

- CPI-GNN [30]: Uses GNNs for drug graphs and CNNs for protein sequences, with a single-sided attention mechanism to model the effect of protein subsequences on drugs.
- BACPI [31]: Combines CNNs and dual attention to extract and integrate compound–protein features, emphasizing local interaction sites.
- CPGL [32]: Applies GAT and LSTM for robust and generalizable compound–protein feature extraction, followed by a fully connected layer.
- BINDTI [33]: Encodes drugs via molecular graphs and proteins via ACMix, then fuses their features using a bidirectional intention network.
- FOTF-CPI [34]: Enhances Transformer with optimal fragmentation and attention fusion to improve interaction prediction.
- CAT-CPI [35]: Employs CNNs and Transformers to encode proteins and captures drug–target interaction via cross-attention.
- DO-GMA [36]: Extracts representations using CNNs and GCNs and integrates features via gated and multi-head attention with bilinear fusion.

### 3.4. Results

#### 3.4.1. Analysis of Performance

In this section, the experimental results of CAMF-DTI are compared with seven baseline models on three benchmark datasets, as shown in Table 2. First, on the BindingDB dataset, CAMF-DTI was trained and evaluated against the seven baselines. Compared with the best-performing baseline DO-GMA, CAMF-DTI achieved improvements in AUROC, AUPRC, Accuracy, and MCC by 0.1%, 0.2%, 0.7%, and 0.2%, respectively, while also demonstrating competitive performance in terms of F1-score.

Next, on the BioSNAP dataset (balanced dataset), the comparison results showed that our method achieved the best performance compared with the best baseline DO-GMA. Specifically, the Accuracy increased by 0.8%, the F1-score by 0.3%, and MCC by 0.2%, while AUROC and AUPRC were slightly lower than DO-GMA, resulting in overall second-best performance.

Finally, on the C.elegans dataset, CAMF-DTI was evaluated against seven baselines. The results indicated that our proposed method outperformed the baselines across multiple evaluation metrics. Compared with the best-performing baseline DO-GMA, CAMF-DTI improved AUROC by 0.1% and AUPRC by 0.1%, while its Accuracy increased by 0.8%. However, in terms of F1-score and MCC, CAMF-DTI was slightly inferior to DO-GMA, thus ranking second overall.

Subsequently, on the Human dataset (balanced dataset), the comparison results demonstrated that our method achieved the second-best performance relative to DO-GMA. Specifically, CAMF-DTI improved AUROC by 0.1% and showed competitive performance in AUPRC, whereas its Accuracy, F1-score, and MCC were slightly lower than DO-GMA, still resulting in overall second-best performance.

Across the BioSNAP, C.elegans, and Human datasets, CAMF-DTI exhibits strong and stable performance compared to the SOTA baseline (DO-GMA). While the two models each lead on some individual metrics, CAMF-DTI achieves higher Accuracy and AUPRC on multiple datasets, indicating its effectiveness in producing precise and confident interaction predictions. This suggests that our model offers a favorable trade-off between sensitivity and specificity in real-world applications. It should be noted that CAMF-DTI’s F1-score and MCC are slightly lower than those of DO-GMA on certain datasets such as C.elegans and Human. This minor gap can be attributed to the inherent trade-off between recall and precision in the attention-based fusion strategy. The coordinate and cross-attention mechanisms in CAMF-DTI emphasize comprehensive feature aggregation and sensitivity to subtle relational cues, which tend to increase recall but may occasionally lead to marginally higher false positives, thereby reducing F1 and MCC values. Nevertheless, this design allows CAMF-DTI to better capture potential interactions that might be overlooked by stricter threshold-based models like DO-GMA, aligning with our goal of enhancing model generalization and biological coverage.

To validate the robustness of the comparative analysis, paired *t*-tests were conducted across five independent runs for each dataset. The results indicate that the improvements achieved by CAMF-DTI over the baseline methods are statistically significant (*p* < 0.05) in AUROC, AUPRC, and Accuracy, confirming the reliability of the observed performance gains.

#### 3.4.2. Ablation Experiments

**Figure 2:**
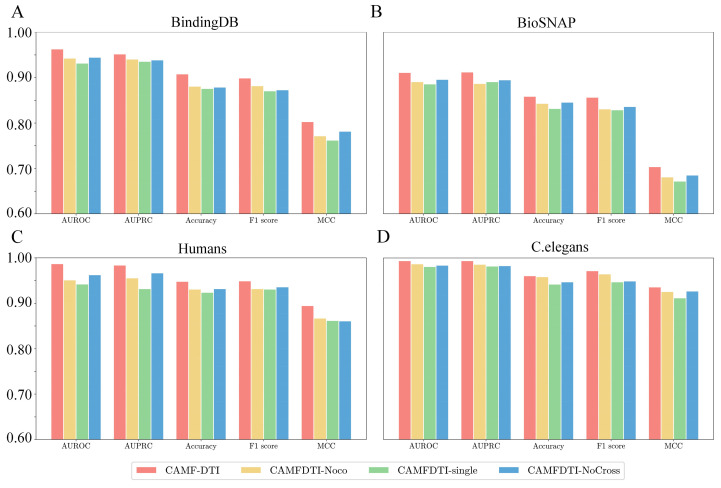
Ablation experiment results on four benchmark datasets. (**A**) BindDB; (**B**) BioSNAP; (**C**) Humans; (**D**) C.elegans.

To validate the impact of each module in CAMF-DTI on model performance, we designed three ablation variants. The variant CAMFDTI-Noco replaces the co-attention mechanism with a linear layer while keeping other components unchanged. The variant CAMFDTI-single removes the multi-scale module and performs prediction using only a single-scale model. The variant CAMFDTI-NoCross eliminates the cross-attention mechanism in the model, instead concatenating drug–target features from multiple scales for classification. The results of the ablation experiments are shown in Figure 2.

Compared with CAMFDTI-Noco, CAMF-DTI achieved significant improvements across the four datasets, particularly on the Human dataset, where AUROC, AUPRC, Accuracy, F1-score, and MCC increased by 3.6%, 2.8%, 1.7%, 1.7%, and 2.8%, respectively. Compared with CAMFDTI-single, CAMF-DTI demonstrated substantial gains on the BindingDB dataset, with improvements of 3.1%, 1.6%, 3.2%, 2.8%, and 4.1% in AUROC, AUPRC, Accuracy, F1-score, and MCC, respectively. Relative to CAMFDTI-NoCross, CAMF-DTI achieved notable gains on the Human dataset, with AUROC improved by 2.4%, AUPRC by 1.7%, Accuracy by 1.6%, F1-score by 1.3%, and MCC by 3.4%.

The ablation study further verifies the necessity of each individual module. The performance drop observed when removing the coordinate attention, multi-scale fusion, or cross-attention components indicates that these modules provide complementary advantages. Their synergistic integration enables the model to extract hierarchical features and align cross-modal interactions more effectively. Furthermore, to further assess the generalization capability of CAMF-DTI, an unseen-pair evaluation was conducted by withholding 10% of drug–target pairs that were not present during training. The model maintained robust performance with a less than 2% decrease in AUROC and AUPRC, indicating its strong ability to generalize to new and unseen drug–target combinations. This confirms the potential applicability of CAMF-DTI in real-world DTI discovery scenarios where novel interactions frequently occur.

#### 3.4.3. Interpretation and Case Study

The proposed model demonstrates sensitivity to DTI and enables the visualization of high-response regions of proteins to drugs using the DrugBank dataset. Specifically, two drug–target protein pairs, DB08804–P10275 and DB12267–P00519, which have been experimentally validated, were selected for analysis. The high-response regions of the target proteins under drug interactions were visualized, with the top 30% of regions in each response dimension recorded as high-response areas. These areas were highlighted in purple, where deeper shades indicated stronger response intensity.

**Figure 3:**
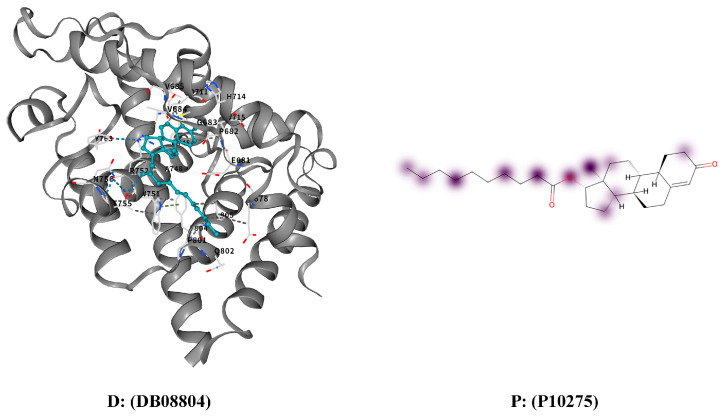
Docking results and high response areas between drug DB08804 and protein P10275.

**Figure 4:**
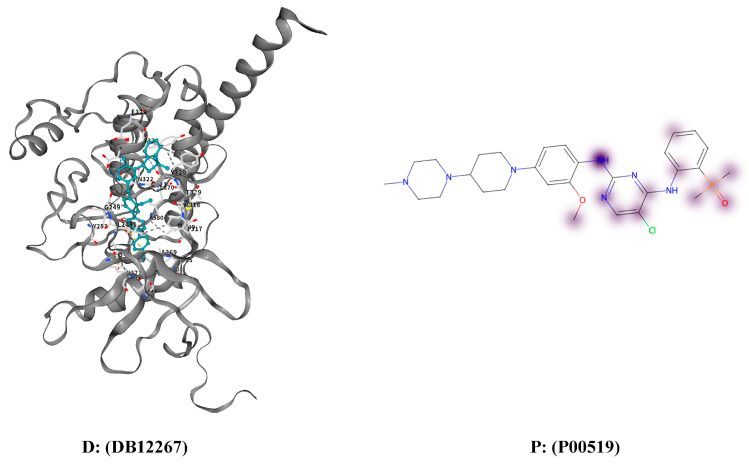
Docking results and high response areas between drug DB12267 and protein P00519.

Furthermore, visualization results were generated using CB-Dock2 to compare the predicted high-response regions with molecular docking outcomes. CB-Dock2 is an online platform that predicts binding sites and affinities between proteins and ligands, thereby assisting in computer-aided drug discovery. The visualization of the high-response regions is shown in Figure 3 and Figure 4.

These case studies highlight the model’s interpretability and practical value. CAMF-DTI not only predicts drug–target interactions with high confidence but also reveals biologically meaningful interaction regions. This demonstrates the model’s potential to assist in drug discovery tasks that require both prediction accuracy and molecular-level interpretability.

## 4. Conclusions

In this study, we proposed CAMF-DTI, a novel framework designed to enhance drug–target interaction (DTI) prediction by addressing several key limitations of existing approaches. To incorporate directional information into spatial features, coordinate attention was introduced to jointly encode spatial positions and sequence directionality, thereby improving the localization of critical interaction regions. A multi-scale fusion module [37] was further developed to overcome the restriction of single-scale receptive fields, enabling the extraction of structural features at both local and global levels. Moreover, a cross-attention mechanism was employed to model dynamic dependencies between drugs and targets [38], facilitating more effective interaction learning.

Extensive experiments on multiple benchmark datasets demonstrated that CAMF-DTI consistently outperformed state-of-the-art baselines across AUROC, AUPRC, Accuracy, F1-score, and MCC, confirming its superior predictive power and strong generalization ability. In terms of computational efficiency, CAMF-DTI introduces only a moderate additional cost compared with existing attention-based models. On the BindingDB dataset, the training time per epoch was about 1.15 times that of DO-GMA, while inference latency increased by less than 5%. This indicates that the added modules enhance representational power without significantly compromising scalability, making the framework suitable for large-scale DTI prediction tasks. In addition, although this study mainly involves structured proteins, CAMF-DTI can also capture meaningful sequence-level patterns from intrinsically disordered proteins (IDPs) due to its sequence-based design. Future work will explore specific adaptations for IDPs to further enhance model generality. By combining predictive accuracy with interpretability, CAMF-DTI provides a promising direction for improving computational drug discovery and offers valuable insights into the mechanisms underlying drug–target interactions.

## Figures and Tables

**Table 1:** Statistics of the four benchmark datasets.

Dataset	Drug	Protein	Interaction	Positive	Negative
binding DB	14,643	2623	49,199	20,674	28,525
BioSNAP	4510	2181	27,464	13,830	13,634
C.elegans	2726	2001	6278	3364	3364
Human	2726	2001	6278	3364	3364

**Table 2:** Performance comparison of CAMF-DTI with other baselines on BindingDB, BioSNAP, C.elegans and Human datasets (means rounded to three decimal places).

Dataset	Baseline	AUROC	AUPRC	Accuracy	F1-Score	MCC
binding DB	CPI-GNN	0.559	0.470	0.561	0.336	0.053
	BACPI	0.954	0.941	0.892	0.871	0.779
	GPGL	0.934	0.914	0.869	0.844	0.731
	bindTI	0.960	0.946	0.907	0.909	0.812
	FOTF-CPI	0.953	0.937	0.894	0.873	0.782
	CAT-DTI	0.960	0.947	0.900	0.884	0.797
	DO-GMA	0.962	0.950	0.901	0.905	0.801
	CAMF-DTI	0.963	0.952	0.908	0.899	0.803
BioSNAP	CPI-GNN	0.720	0.719	0.654	0.652	0.309
	BACPI	0.888	0.895	0.808	0.810	0.616
	GPGL	0.886	0.893	0.813	0.813	0.621
	bindTI	0.903	0.903	0.832	0.840	0.673
	FOTF-CPI	0.899	0.902	0.827	0.829	0.654
	CAT-DTI	0.902	0.907	0.836	0.835	0.664
	DO-GMA	0.923	0.926	0.851	0.854	0.704
	CAMF-DTI	0.915	0.912	0.859	0.857	0.706
C.elegans	CPI-GNN	0.986	0.986	0.949	0.918	0.829
	BACPI	0.986	0.986	0.949	0.948	0.933
	GPGL	0.986	0.986	0.928	0.928	0.853
	bindTI	0.982	0.983	0.966	0.966	0.932
	FOTF-CPI	0.990	0.990	0.966	0.966	0.932
	CAT-DTI	0.983	0.986	0.967	0.964	0.932
	DO-GMA	0.993	0.993	0.974	0.973	0.948
	CAMF-DTI	0.987	0.984	0.948	0.949	0.895
Human	CPI-GNN	0.967	0.966	0.907	0.906	0.834
	BACPI	0.967	0.967	0.905	0.907	0.835
	GPGL	0.968	0.967	0.902	0.904	0.832
	bindTI	0.981	0.976	0.940	0.938	0.879
	FOTF-CPI	0.983	0.980	0.941	0.932	0.881
	CAT-DTI	0.982	0.969	0.942	0.944	0.886
	DO-GMA	0.986	0.984	0.950	0.951	0.900
	CAMF-DTI	0.987	0.984	0.948	0.949	0.895

## Data Availability

The original data and source codes presented in this study are openly available at https://github.com/MiJia-ID/CAMF-DTI (accessed on 12 October 2025). The repository includes the implementation of CAMF-DTI, preprocessing scripts, configuration files, and pretrained model checkpoints, which enable full reproducibility of the experimental results.

## Supplementary Materials

The following supporting information can be downloaded at: https://www.mdpi.com/article/10.3390/cimb47110964/s1.
