# Supplementary material for: CAMF-DTI: Enhancing Drug–Target Interaction Prediction via Coordinate Attention and Multi-Scale Feature Fusion
Source: Curr Issues Mol Biol. 2025 Nov 20;47(11):964. doi: 10.3390/cimb47110964 (PMC12651166; doi:10.3390/cimb47110964)
Supplement: Supplementary file 1 [file cimb-47-00964-s001.zip › cimb-3953636-supplementary.pdf]

Table S1

| Category | Descriptor Name | Description |
|---|---|---|
| **Drug features (atom-level)** | Atom type | Element symbol (C, N, O, etc.) |
| | Degree | Number of directly bonded neighbors |
| | Formal charge | Atomic charge state |
| | Hybridization | sp, $sp^2$, $sp^3$ configuration |
| | Aromaticity | Binary indicator of aromatic structure |
| | Chirality | R/S configuration |
| | Implicit hydrogen count | Number of attached hydrogens |
| | Ring membership | Whether an atom belongs to a ring |
| **Protein features (residue-level)** | Amino acid index | Integer mapping of 22 amino acid types |
| | Hydrophobicity | Scaled physicochemical property |
| | Polarity | Binary indicator of polar residues |
| | Charge | Residue charge type (positive/negative/neutral) |
| | Molecular weight | Standard residue mass |
